# Fine mapping and candidate gene analysis of *CRA8.1.6*, which confers clubroot resistance in turnip (*Brassica rapa* ssp. *rapa*)

**DOI:** 10.3389/fpls.2024.1355090

**Published:** 2024-05-17

**Authors:** Xiaochun Wei, Shixiong Xiao, Yanyan Zhao, Luyue Zhang, Ujjal Kumar Nath, Shuangjuan Yang, Henan Su, Wenjing Zhang, Zhiyong Wang, Baoming Tian, Fang Wei, Yuxiang Yuan, Xiaowei Zhang

**Affiliations:** ^1^ Institute of Vegetables, Henan Academy of Agricultural Sciences, Graduate T&R Base of Zhengzhou University, Zhengzhou, Henan, China; ^2^ School of Agricultural Sciences, Zhengzhou University, Zhengzhou, Henan, China; ^3^ Department of Genetics and Plant Breeding, Bangladesh Agricultural University, Mymensingh, Bangladesh

**Keywords:** turnip, clubroot, fine mapping, C-terminal, CRA08-InDel

## Abstract

Clubroot disease poses a significant threat to *Brassica* crops, necessitating ongoing updates on resistance gene sources. In F_2_ segregants of the clubroot-resistant inbred line BrT18-6-4-3 and susceptible DH line Y510, the genetic analysis identified a single dominant gene responsible for clubroot resistance. Through bulk segregant sequencing analysis and kompetitive allele-specific polymerase chain reaction assays, *CRA8.1.6* was mapped within 110 kb (12,255–12,365 Mb) between markers L-CR11 and L-CR12 on chromosome A08. We identified *B raA08g015220.3.5C* as the candidate gene of *CRA8.1.6*. Upon comparison with the sequence of disease-resistant material BrT18-6-4-3, we found 249 single-nucleotide polymorphisms, seven insertions, six deletions, and a long terminal repeat (LTR) retrotransposon (5,310 bp) at 909 bp of the first intron. However, the LTR retrotransposon was absent in the coding sequence of the susceptible DH line Y510. Given the presence of a non-functional LTR insertion in other materials, it showed that the LTR insertion might not be associated with susceptibility. Sequence alignment analysis revealed that the fourth exon of the susceptible line harbored two deletions and an insertion, resulting in a frameshift mutation at 8,551 bp, leading to translation termination at the leucine-rich repeat domain’s C-terminal in susceptible material. Sequence alignment of the CDS revealed a 99.4% similarity to *Crr1a*, which indicate that *CRA8.1.6* is likely an allele of the *Crr1a* gene. Two functional markers, CRA08-InDel and CRA08-KASP1, have been developed for marker-assisted selection in CR turnip cultivars. Our findings could facilitate the development of clubroot-resistance turnip cultivars through marker-assisted selection.

## 1 Introduction

Turnip (*Brassica rapa* L. spp. *rapifera*) is a vegetable belonging to the genus *Brassica*. It originated in Afghanistan, Pakistan, Transcaucasia (part of Asia), and the Mediterranean. The Asian turnip variety is predominantly distributed in China and western Japan (Pu et al., 2018). Clubroot disease is caused by *Plasmodiophora brassicae* (*P. brassicae*) and has been reported in numerous countries (Chai et al., 2014). It affects plants in the *Brassicaceae* family, such as Chinese cabbage, turnips, radish, cauliflower, and mustard (Howard et al., 2010). *P. brassicae* first infects root hairs by free primary spores and then releases secondary zoospores to invade the cortex (Kageyama and Asano, 2009). This results in gradual swelling of the roots in affected plants, hindering nutrient absorption and ultimately leading to wilting and death of the entire plant. Dormant spores of *P. brassicae* can remain active in the soil for up to 20 years, posing a long-term threat to cruciferous plants (Dixon, 2009). Clubroot diseases tend to exacerbate annually, making control challenging through chemical, biological, and agricultural means. From an ecological standpoint, developing resistant varieties through breeding is a promising solution (Piao et al., 2009). Zhang et al. (2024) successfully identified a CR gene, facilitating CR-resistant breeding in *B. oleracea*.

Single-nucleotide polymorphisms (SNPs) represent a prevalent form of DNA variation across the genome, offering advantages such as high throughput and seamless integration. Consequently, they find wide applications in various fields, such as disease treatment, drug development, and plant breeding. The kompetitive allele-specific polymerase chain reaction (PCR) (KASP) technique is a precise allele-specific PCR method capable of accurately identifying SNPs by matching terminal primer bases. KASP technology is renowned for its time efficiency, reduced error rates, and cost-effectiveness in genotyping, providing flexibility for genotyping multiple samples with minimal SNP loci (Gouda et al., 2021).

Significant progress has been made in developing clubroot-resistant genotypes. European fodder turnips (*Brassica rapa* L. spp. *rapifera*) (AA, 2n = 20) ‘ECD01-04,’ ‘Gelria R,’ ‘Siloga,’ ‘Debra,’ and ‘Milan White’ (Eckholm, 1993; Elke et al., 2009) have emerged as a widely utilized resistant source, successfully integrated into CR breeding programs in Chinese cabbage and canola (Piao et al., 2009). To date, 36 clubroot-resistant loci have been identified across *Brassica* species, including *B. rapa*, *B. oleracea*, *B. napus*, and *B. nigra* (Hasan et al., 2021; Pang et al., 2022). Among these loci, the majority are found on chromosomes A03 and A08. Chromosome A03 harbors 19 resistance loci, encompassing *CRa*, *CRb*, *CRd*, *CRq*, *CRk*, *Rcr1*, *Rcr2*, *Rcr4*, *Rcr5*, *pbBa3.1*, *pbBa3.2*, *pbBa3.3*, *CR6b*, *CRb^kato^*, *BraA.CRa*, *BraA.CRc*, *BraA3PSX.CRa/b^kato^1.1*, *BraA3PSX.CRa/b^kato^1.2*, and *Crr3* (Etsuo et al., 1998; Hirai et al., 2004; Piao et al., 2004; Ueno et al., 2012; Chen et al., 2013; Chu et al., 2014; Sun et al., 2016; Yu et al., 2017; Pang et al., 2018; Huang et al., 2019). On chromosome A08, nine resistance loci are identified, namely, *CRs*, *Crr1*, *Rcr3*, *Rcr9*, *PbBa8.1*, *qBrCR38-2*, *RCr9^wa^*, *PbBrA08^Banglim^*, and *BraACRb* (Hirani et al., 2018; Laila et al., 2019; Md. Masud et al., 2020; Yu et al., 2022). Additionally, chromosome A01 hosts three loci, namely, *CR6a*, *Crr2*, and *PbBa1.1* (Suwabe et al., 2006). Chromosome A02 carries two loci, namely, *CRc* and *Rcr8* (Yu et al., 2017), whereas resistance loci *CrrA5*, *Crr4*, and q*BrCR38-1* are located on chromosomes A05, A06, and A07, respectively (Suwabe et al., 2006; Nguyen et al., 2018; Zhu et al., 2019). The collection of resistance loci appears to be extensive; however, only *Crr1a*, *CRa*, and their alleles have been cloned (Ueno et al., 2012; Hatakeyama et al., 2013; Yang et al., 2022). These cloned genes have been found to contain important components of effector-induced immunity, TIR-NBS-LRR [Toll/interleukin-1 (IL-1) receptor–like nucleotide binding site, leucine-rich repeat] protein domain family (Mariana et al., 2002).

*CRA8.1.6* was finely mapped and cloned from the F_2_ population, resulting from a cross between the resistant inbred line BrT18-6-4-3 and the susceptible DH line Y510. Our investigation aimed to elucidate the mechanisms underlying the loss of resistance by analyzing the structure of candidate genes in the susceptible line. Gene sequence alignment revealed a frameshift mutation in the susceptible line Y510, resulting in translation termination at 8,551 bp. This mutation altered the C-terminal leucine-rich repeat (LRR) domain, leading to a loss of resistance. The disease index survey demonstrated a significantly lower disease index in overexpression transgenic *Arabidopsis* than in wild-type *Arabidopsis*. The results of the target gene expression revealed a significant increase in the relative expression of *CRA8.1.6* in the T_2_ generation of *Arabidopsis* compared to the wild-type. This indicates that the conferred *CRA8.1.6* gene is indeed a clubroot-resistant gene. Functional markers, CRA08-InDel and CRA08-KASP1, linked to *CRA8.1.6* were successfully developed and validated. These markers represent valuable resources for marker-assisted selection in breeding CR cultivars against *P. brassicae*.

## 2 Materials and methods

### 2.1 Plant materials

In the current study, F_1_ and F_2_ populations were developed by crossing the clubroot-resistant turnip inbred line BrT18-6-4-3 (P_1_) with the susceptible Chinese cabbage DH line Y510 (P_2_) followed by the selfing of F_1._ Parental lines, F_1_, and F_2_ populations, were inoculated with *P. brassicae* race four isolate ‘XY-2’ (Yuan et al., 2017). The F_2_ population was cultivated between September 2021 and November 2021, and the inheritance pattern of the resistant gene was determined using a Chi-square test (*χ* ^2^). Furthermore, 26 disease-resistant and 29 susceptible lines were used to analyze mutations in the candidate gene (**Supplementary Table 1**). All materials used in this study were provided by the Institute of Vegetables, Henan Academy of Agricultural Sciences.

### 2.2 Estimation of disease index

The disease index was assessed by inoculating the plants on the 20th day after sowing using a root irrigation method with a concentration of 10^7^ spores/mL of *P. brassicae* suspension (Luo et al., 2014). AKIMEKI and ECD05 were utilized for disease index estimation as controls for disease resistance and susceptibility, respectively (Matsumoto et al., 2012). The inoculated plants were maintained at 22°C ± 2°C and 16-h/8-h (light/dark) photoperiod (Yuan et al., 2021). Samples were collected and immediately frozen in liquid nitrogen and then stored at −80°C for subsequent use. Root samples from resistant and susceptible materials were collected at different time points: 0 days after inoculation (DAI; indicating no infection), 3 DAI (representing cortical infection), 9 DAI (indicating early onset of disease), and 20 DAI (indicating late onset of disease). One-centimeter-long root segments were cut from the junction of the rhizome and fixed with Formalin-Aceto-Alcohol (FAA) solution (containing 5 mL of acetic acid + 5 mL of formalin + 50 mL of 95% alcohol + 35 mL of sterile water) for 24 h. After fixation with FAA, the roots were rinsed with sterile water, dried with absorbent paper, and stained with 0.5% fluorescent pink for 3 h. The infected roots were observed under a light microscope (Li et al., 2022).

### 2.3 Bulked segregant analysis by resequencing

DNA was extracted from resistant and susceptible parents, along with 30 highly resistant (DI = 0; R-pool) and susceptible F_2_ plants (DI = 5–7; S-pool) (**Supplementary Table 1**) following the cetyl trimethyl ammonium bromide method. Bulked segregant analysis (BSA) sequencing (BSA-seq) was performed on four DNA pools (Huang et al., 2017), and sequencing was conducted using the Illumina HiSeq platform, with analysis carried out using NovoGene (http://www.novogene.com). The raw reads underwent quality control trimming to remove low-quality paired reads with the adapter and retained clean reads. The clean reads from the four DNA bulks were combined using BWA software (V0.7.17) and compared with *B. rapa* (Chiifu-401) genome version 3.0 (http://brassicadb.org/brad/index.phpM) (Kathiresan et al., 2014). SAMtools software (V1.3.1) was employed to identify SNPs and insertion/deletion (InDel) variations between the R and S pools. The ΔSNP index for all genomic positions in the R and S libraries was calculated (**Supplementary Table 2**) using a sliding window analysis with a window width of 1 Mb and a sliding window step size of 10 kb.

### 2.4 Marker analysis and linkage map construction

Sequences of the genes were downloaded from the *Brassica* database (http://brassicadb.org/brad/downloadOverview.php), and the SNPs of candidate genes were validated. Thirty-five pairs of primers for KASP markers were designed using DNAMAN software (V6.0) to verify the parents and F_2_ population (**Supplementary Table 3**), and, ultimately, 21 pairs of polymorphic primers were selected (Xiang et al., 2007).

The KASP markers were utilized for genotyping the F_2_ population consisting of 98 individuals. Linked loci were identified using JoinMap4.0 to form linkage groups (Voorrips, 2002), and a genetic linkage map was constructed according to the Kosambi mapping function (Kosambi, 1944).

An additional large F_2_ population of 1,489 individuals was planted for recombination selection, from which 438 susceptible individuals were selected and used to map candidate genes.

### 2.5 Cloning and sequencing of the *CRA8.1.6* gene

DNA and cDNA sequences of *CRA8.1.6* candidate genes were cloned from the parents using Phanta Super-Fidelity DNA polymerase Mix (Vazyme, Nanjing, China) (**Supplementary Table 4**). Standard PCR was conducted with 30-s denaturation at 98°C followed by 35 cycles of 98°C for 10 s, 58°C for 5 s, and 72°C for 60 s.

PCR products were sequenced by Sangon Biotech Co., Ltd. (Zhengzhou, China) and analyzed using DNAMAN software (V6.0). The complete coding sequence (CDS) of the *CRA8.1.6* gene from BrT18-6-4-3 and Y510 has been submitted to GenBank under the accession number AB605024.1. Protein’s physical and chemical properties were determined with Expasy (http://web.expasy.org/protparam/).

### 2.6 Quantitative real-time PCR and semi-quantitative RT-PCR analyses

Total RNA was extracted from root samples collected at 0 DAI, 3 DAI, 9 DAI, and 20 DAI of BrT18-6-4-3 and Y510 lines using the RNA Prep Pure Plant Kit (Beijing Day, China) (Wei et al., 2021). Single-stranded cDNA was synthesized using Trans-script after a two-step removal of genomic DNA (gDNA) (Trans, Beijing, China).

The quantitative real-time PCR (qRT-PCR) was performed in a Roche Light Cycler 480-II system (Roche Applied Sciences, Beijing, China). *GAPDH* was used as an internal reference gene to calculate relative expression levels using the 2^−ΔΔCt^ method (Livak and Schmittgen, 2001). *B. rapa* sequence information was utilized to design RT-PCR primers. Standard PCR was conducted by denaturing for 5 min at 94°C, followed by 30 s at 94°C, 30 s at 55°C, and 60 s at 72°C for 25 cycles. PCR products were visualized after electrophoresis on a 1% agarose gel (**Supplementary Table 4**).

### 2.7 Vector construction and transformation

Specific full-length primers were designed to incorporate restriction sites and protective bases. This was achieved using the plasmid DNA of the target gene as a template. The PCR amplification procedure was conducted as follows: 95°C for 5 min; 30 cycles of 95°C for 30 s, 50°C for 45 s, and 72°C for 258 s; followed by 72°C for 10 min; and 16°C for 30 min.

The purified PCR product was digested with pBWA (V) HS expression vector (Bio Run, Hu Bei, China), and the digested product was further purified and ligated with the vector using T4 ligase to obtain the recombinant plasmid pBWA (V) HS-BrT18-6. The ligation product was then transformed into competent DH5α cells and PCR-screened positive clones. After sequencing and verification, *Arabidopsis thaliana* was transformed using dip method (Hamann et al., 2002). The resulting T_0_ transgenic *A. thaliana* was identified (Pang et al., 2022), and positive plants were selected for future planting to obtain T_2_ generation seeds.

### 2.8 Inoculation of overexpression transgenic *A. thaliana* and identification of candidate gene

The T_2_ generation seeds of *A. thaliana* were sown on a medium containing hygromycin to select homozygous strains based on positive reactions on specific media (Tang, 2007). T_2_ generation *A. thaliana* seeds were planted (Suwabe et al., 2006). The *P. brassicae* strain used in this study was obtained from a clubroot-infected Chinese cabbage field (*B. rapa*) in Xinye County, Henan Province, China (113.97°E, 35.05°N), and the Williams system was used to confirm the *P. brassicae* strain as race 4 (Yuan et al., 2017).

Twenty-five-day-old transgenic and wild-type *A. thaliana* were inoculated with *P. brassicae*. Similarly, another set was inoculated with fresh water without any *P. brassicae* spores, which served as the control group. After 30 days of inoculation, the wild-type and transgenic *A. thaliana* were examined in control and experimental groups. The frequency of clubroot disease and expression of candidate genes were observed (Keita et al., 2012).

## 3 Results

### 3.1 Phenotypic and genetic analyses and cytological observation

After 30 days of infection with *P. brassicae*, all F_1_ individuals resisted clubroot disease. We inoculated two fractions of the F_2_ population with *P. brassicae* to account for the nature of disease-resistance gene action. In a small fraction of the F_2_ population, 341 plants were resistant and 132 were susceptible, whereas, in a large fraction, 1,134 plants were resistant and 335 were susceptible. Both F_2_ populations exhibited a 3:1 segregation, indicating Mendelian genetics for a single dominant gene (**Table 1**).

**Table 1:** Genetic analysis of clubroot resistance and susceptibility in F_2_ populations of the cross between BrT18-6-4-3 and Y510.

Experiment	Population size	Resistant plants	Susceptible plants	Segregation	χ^2^	χ^2^_(0.05)_
P_1_ (BrT18-6-4-3)	12	12	0	–	–	–
P_2_ (Y510)	12	0	12	–	–	–
F_1_	48	48	0	–	–	–
F_2_-small	473	341	132	3:1	2.13	3.84
F_2_-large	1,489	1,134	355	3:1	1.07	3.84

Microscopic examination revealed abundant spores attached to root hairs in all test plants at 1 DAI, indicating sufficient spores to initiate root infection. However, no cortical infection was detected in the resistant line (BrT18-6-4-3) at 1 DAI, 2 DAI, and 3 DAI or in the control group. In contrast, root hair infection was observed in the susceptible line (Y510) at 1 DAI, with cortical infection and free spores in epidermal cells observed at 3 DAI. No such symptoms were observed in the susceptible lines of the control group.

**Figure 1:**
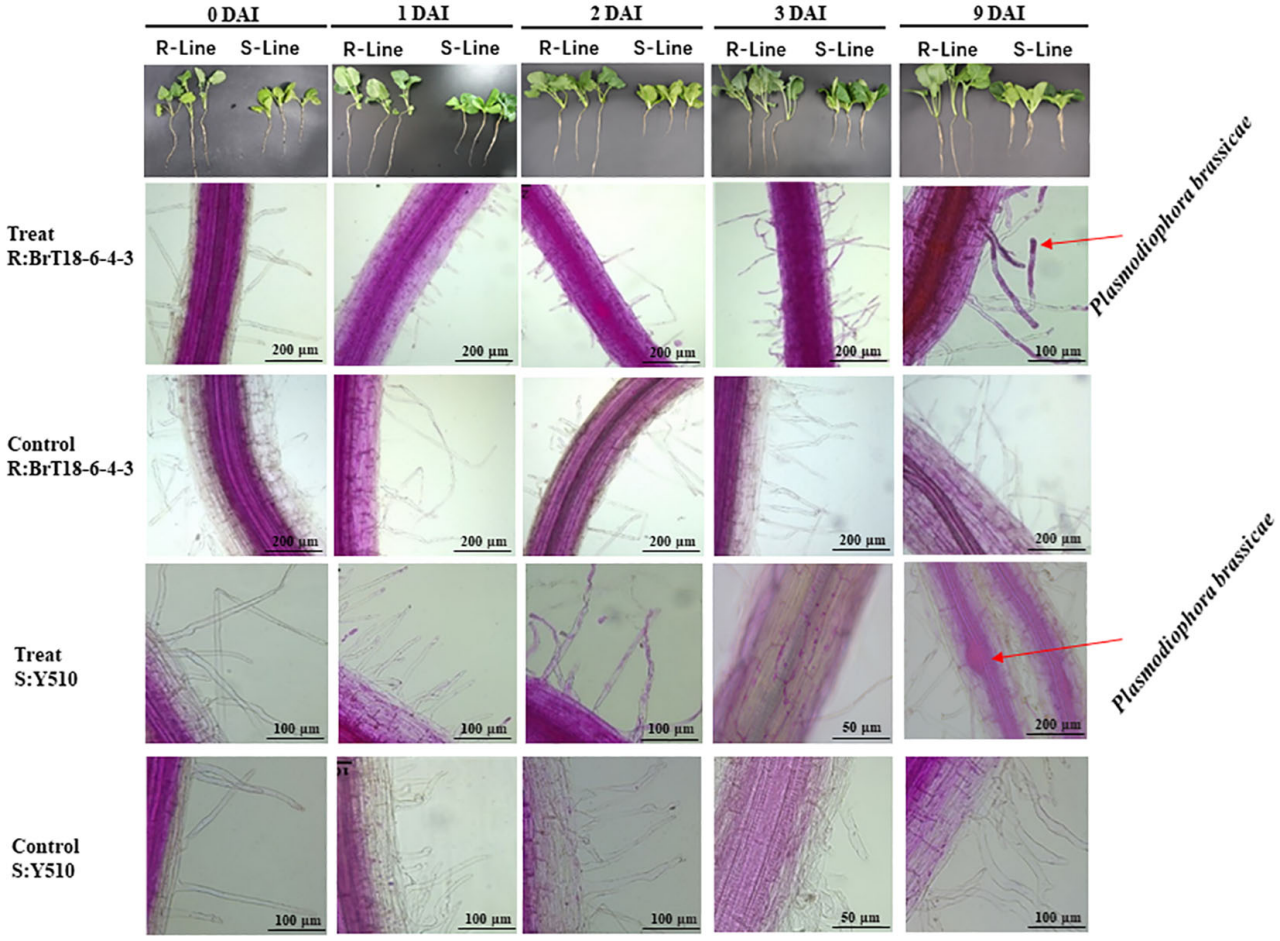
Comparison of root infection status between R-line (BrT18-6-4-3) and S-line (Y510) of control and *P. brassicae* inoculated groups. BrT18-6-4-3 initiated root hair infection at 1 DAI, and few spores were present in cortical cells at 9 DAI. Y510 showed root hair infection at 1 DAI and cortical infection at 3 DAI to 9 DAI, with many spores in cortical cells at 9 DAI. Bar = 200 μm, 100 μm, and 50 μm.

At 9 DAI, the roots of the resistant line appeared normal, whereas the roots of the susceptible line were slightly swollen. Numerous spores were found in the swollen root cortical cells of the susceptible line. In contrast, only a few spores were observed in the cortical cells of the resistant line (**Figure 1**), indicating cortical infection occurred between 3 DAI and 9 DAI.

### 3.2 Mapping of *CRA8.1.6* QTL

BSA-seq analysis generated 36,416,118 and 38,958,860 clean reads from the R and S pools, respectively (**Supplementary Table 2**). These reads were aligned to the *B. rapa* genome, identifying 218,150 SNPs and 53,040 InDels between the R and S pools. △ (SNP index) for each locus was calculated using sliding window analysis. Subsequently, a new quantitative trait locus (QTL) called *CRA8.1.6* was identified on chromosome A08, likely between 11.04 Mb and 16.10 Mb (**Supplementary Figure 1**).

**Figure 2:**
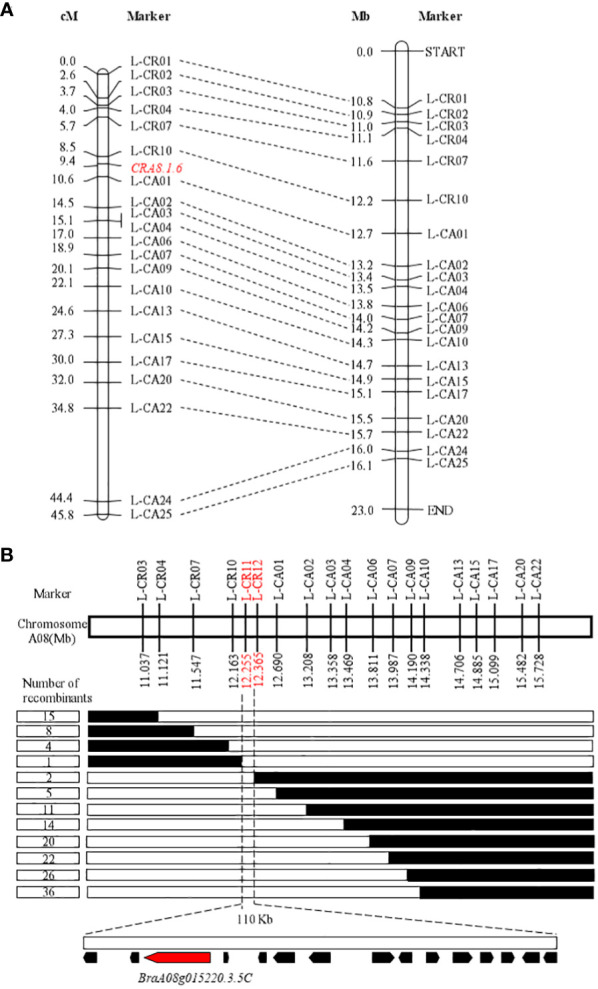
Initial and fine mapping of the *CRA8.1.6* in Chinese Turnip. **(A)** Preliminary mapping of the area *CRA8.1.6*. The left side depicts the *CRA8.1.6* genetic map, with marker distance indicated in cM. The corresponding physical map is also shown on the right, with units in megabases (Mb). **(B)** The *CRA8.1.6* gene was delineated between markers L-CR11 and L-CR12, spanning an estimated length of 110 kb. Fifteen genes were annotated on the basis of the reference genome sequence (Chiifu-401-42). Each genotype is represented, with white indicating the homozygote and black representing the heterozygote. The number of recombinants is displayed in the boxes to the left of the map.

Thirty-five KASP markers were created on the basis of SNP variants within the candidate interval and polymorphism between the resistant (BrT18-6-4-3) and susceptible (Y510) lines, with 21 markers selected for genotyping of 98 F_2_ plants to develop markers for chromosome A08. Within the reported *CRA8.1.6* QTL, one recombinant individual was found each for markers L-CR10 and L-CA01 with a genetic distance of 0.9 cM and 1.2 cM, respectively (**Figure 2A**). The order of markers on the genetic map was consistent with the physical map.

To refine the *CRA8.1.6* QTL, 438 susceptible homozygous F_2_ individuals were screened for polymorphisms between markers L-CR03 and L-CA22, identifying 36 recombinants. All 51 recombinants were further genotyped using markers L-CR10 and L-CA01, identifying nine recombinants. Five KASP markers were developed, two of which showed polymorphism. Ultimately, a candidate gene for the *CRA8.1.6* QTL was determined at a position (12,255–12,365 Mb) on chromosome A08 marked by markers L-CR11 and L-CR12 (**Figure 2B**).

### 3.3 Candidate gene analysis

**Figure 3:**
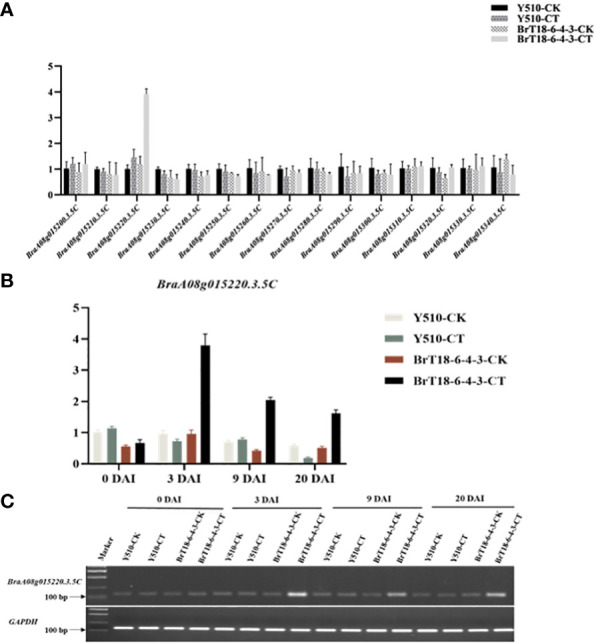
Gene expression of two parents in experimental and control groups at different periods. **(A)** qRT-PCR analysis of 15 candidate genes. **(B)** The expression of *BraA08g015220.3.5c* across different periods. **(C)** Semi-quantitative RT-PCR of *CRA8.1.6* (*BraA08g015220.3.5c*), with *GAPDH* served as the internal control.

Detailed mapping of the *CRA8.1.6* QTL region revealed 15 genes, with *BraA08g015220.3.5C* being the sole gene encoding the TIR-NBS-LRR domains (**Table 2**). We utilized qRT-PCR to examine the expression of the hypothesized candidate gene. Among the 15 genes, only *BraA08g015220.3.5C* exhibited differential expression between the parental lines (**Figure 3A**). The expression of the *BraA08g015220.3.5C* gene was analyzed across various periods. The *BraA08g015220.3.5C* gene exhibited a four-fold increase in expression in the inoculated resistant line (BrT18-6-4-3) compared to the non-inoculated resistant line and both the inoculated and non-inoculated susceptible (Y510) lines at 3 DAI. Similarly, at 9 DAI, there was a two-fold increase in expression in the inoculated resistant line compared to the other lines (**Figure 3B**). The trend of gene expression analyzed by RT-PCR was consistent with that of qPCR (**Figure 3C**). Hence, the *BraA08g015220.3.5C* gene was identified as a potential candidate gene responsible for imparting resistance against clubroot disease.

**Table 2:** Annotated genes in the candidate region of *CRA8.1.6*.

Gene name	Gene position	Homolog gene	Gene function
*BraA08g015200.3.5C*	12,252,676–12,258,432	*AT4G21910*	MATE efflux family protein
*BraA08g015210.3.5C*	12,269,419–12,271,510	Unknown	Gag-polypeptide of long terminal repeat (LTR) copia-type
*BraA08g015220.3.5C*	12,271,230–12,287,495	*AT3G25510*	Disease-resistance protein (TIR-NBS-LRR class) family protein
*BraA08g015230.3.5C*	12,289,217–12,290,401	*AT4G22030*	F-box protein with a domain protein
*BraA08g015240.3.5C*	12,298,469–12,300,228	*AT4G22080*	Root hair–specific 14
*BraA08g015250.3.5C*	12,302,519–12,306,933	*AT4G22100*	Beta glucosidase 2
*BraA08g015260.3.5C*	12,310,890–12,314,574	*AT4G22120*	Calcium-permeable stretch-activated cation channel
*BraA08g015270.3.5C*	12,326,130–12,329,824	*AT4G22130*	STRUBBELIG-receptor family 8
*BraA08g015280.3.5C*	12,329,820–12,332,255	*AT4G22140*	Encodes a chromatin remodeling factor that regulates flowering time
*BraA08g015290.3.5C*	12,334,419–12,335,973	*AT4G22220*	Encodes a mitochondrial protein accepting sulfur and iron to build a transient Fe-S cluster
*BraA08g015300.3.5C*	12,336,747–12,338,591	*AT4G05530*	Encodes a peroxisomal member of the short-chain dehydrogenase/reductase family of enzymes
*BraA08g015310.3.5C*	12,344,064–12,346,459	*AT4G22360*	SWIB complex BAF60b domain-containing protein
*BraA08g015320.3.5C*	12,347,226–12,348,876	*AT4G12590*	ER membrane protein complex subunit-like protein
*BraA08g015330.3.5C*	12,356,313–12,357,823	*AT4G11580*	Methylesterase PCR A
*BraA08g015340.3.5C*	12,358,221–12,360,158	LOC103834248	Pectinesterase

### 3.4 Sequence variation of candidate genes between resistant and susceptible lines

**Figure 4:**
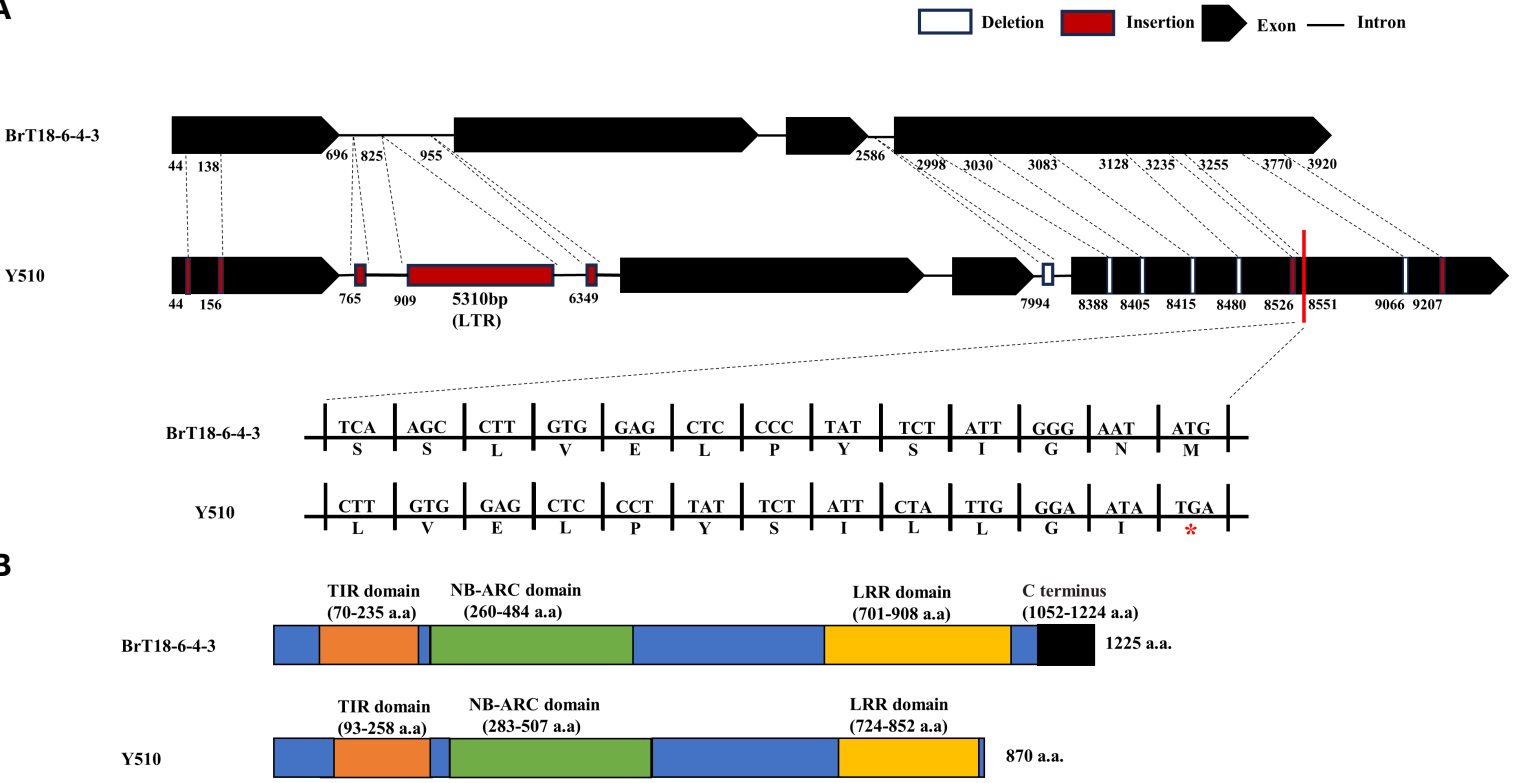
*CRA8.1.6* gene structure, amino acid analysis, and protein structure prediction. **(A)** *CRA8.1.6* comprises four exons and three introns. Gene sequences in the disease-susceptible material exhibited 249 SNPs, seven insertions, and six deletions, including 5,310 bp insertions at 909 bp. INS, insertion; DEL, deletion. **(B)** A frameshift mutation caused premature termination at 870 amino acids of exon four, disrupting the LRR domain in the C-terminal region.

PCR products of the *BraA08g015220.3.5C* gene were utilized to determine gDNA and CDS. Specific primers amplify disease-resistant (BrT18-6-4-3) and susceptible (Y510) lines. In the resistant line, the genome and CDS sequences of *BraA08g015220.3.5C* were 4295 bp and 3675 bp, respectively. Conversely, in the susceptible parent, the genome and CDS sequences were 9586 bp and 3644 bp, respectively (**Supplementary Figure 2**). Conserved domain analysis unveiled several domains within the *BraA08g015220.3.5C* gene, including a Toll and IL-1 domain (TIR, amino acids 70–235), a nucleotide binding site (NBS-ARC, amino acids 260–484), and an LRR (amino acids 701–908). Compared to disease-resistant and susceptible materials, we identified 249 SNPs, seven insertions, and six deletions in the susceptible line. Among these mutations, the insertion of the long terminal repeat (LTR) retrotransposon does not influence the *CRA8.1.6* function, as it does not alter the CDS sequence (**Figure 4A**, **Supplementary Figure 2**). The sequencing results revealed six non-synonymous mutations that did not influence the resistance of the wild, because these mutations were contained in both resistant and susceptible materials (**Supplementary Figures 3**, **4**). Furthermore, sequence alignment analysis revealed that the fourth exon of the susceptible line harbored two deletions and an insertion, leading to premature termination of the C-terminus and destruction of the LRR domain (**Figure 4B**). This premature termination consequently led to the loss of *CRA8.1.6* function, rendering Y510 susceptible to clubroot disease.

**Figure 5:**
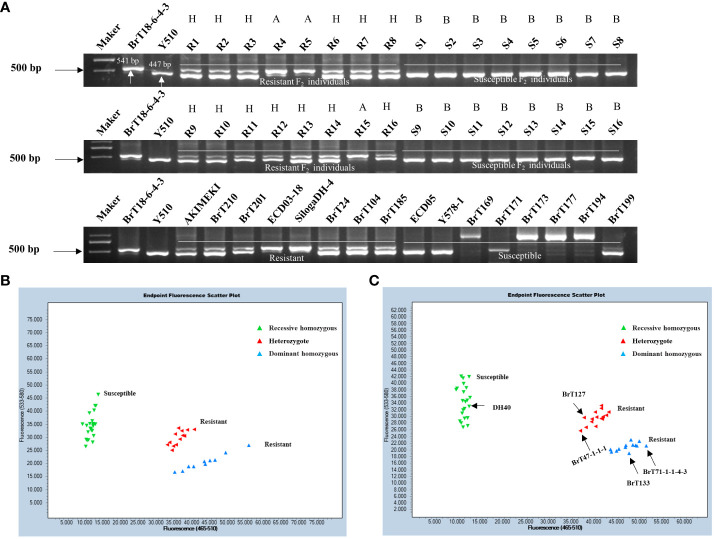
Validation of functional markers. **(A)** Validation of the CRA08-Indel marker in F_2_ individuals and natural populations. **(B)** Validation of the CRA08-KASP1 marker in F_2_ individuals. **(C)** Validation of the CRA08-KASP1 marker in 24 disease-resistant (AKIMEKI, ECD03-18, ECD04-1, ECD04-15, SilogaDH-4, SilogaDH-5, BrT22, BrT24, BrT25, BrT30-2-1-3, BrT81, BrT104, BrT114DH-1, BrT114DH-2, BrT114DH-3, BrT185, BrT201, BrT210, BrT238, BrT242, BrT243, WJ2-5-1, and DH40) and 26 disease-susceptible materials (ECD05, R16, BrT121, BrT127, BrT47-1-1-1, BrT130, BrT131, BrT133, BrT165, BrT169, BrT171, BrT71-1-1-4-3, BrT177, BrT193, BrT194, BrT199, BrT221, BrT222, BrT223, BrT224, BrT225, BrT226, BrT228, BrT229, BrT232, and Y578-1). Note: A, dominant homozygous; B, recessive homozygous; and H, heterozygote.

Using the sequence variation observed in the fourth exon between the parental lines, we designed upstream and downstream primers for 2,855 bp and 3,395 bp, respectively. Functional markers, including CRA08-InDeL (**Supplementary Table 4**), were also developed to distinguish individuals of BrT18-6-4-3, Y510, and F_2_ populations. The amplified fragment size was 541 bp in the resistant materials and 447 bp in the susceptible materials. The results indicated complete concordance between the phenotypes of resistant, susceptible, and heterozygous F_2_ plants with their respective genotypes. This suggests that CRA08-InDel is a functional marker capable of accurately distinguishing clubroot-resistance phenotypes within segregating populations (**Figure 5A**). Within a natural population, the genotypes of disease-resistant materials consistently matched their phenotypes. However, the identification of susceptible materials was not sufficiently consistent. Hence, we sequenced materials that did not match this marker and identified a trait-associated SNP at 3,209 bp. Subsequently, we developed the KASP functional marker CRA08-KASP1 (**Supplementary Table 4**). Upon screening F_2_ populations with this marker, we observed that the expected amplified products for resistant, susceptible, and heterozygous F_2_ individuals perfectly matched the anticipated results. This suggests that the marker CRA08-KASP1 segregated in the F_2_ population with a clubroot-resistant phenotype (**Figure 5B**). The CRA08-KASP1 markers were also screened for 24 disease-resistant and 26 disease-susceptible materials. Among these, the phenotype and genotype of all 23 resistant materials were matched entirely, whereas the phenotype and genotype of 22 susceptible materials were matched. However, only five materials (DH40, BrT127, BrT47-1-1-1, BrT133, and BrT71-1-1-4-3) did not correspond with the genotype (**Figure 5C**). In our future research, we will clone the genes of these five non-matching materials identified by the CRA08-KASP1 marker to explore other variations that might cause clubroot disease.

### 3.5 Construction of an overexpression vector and the development of transgenic *Arabidopsis*

**Figure 6:**
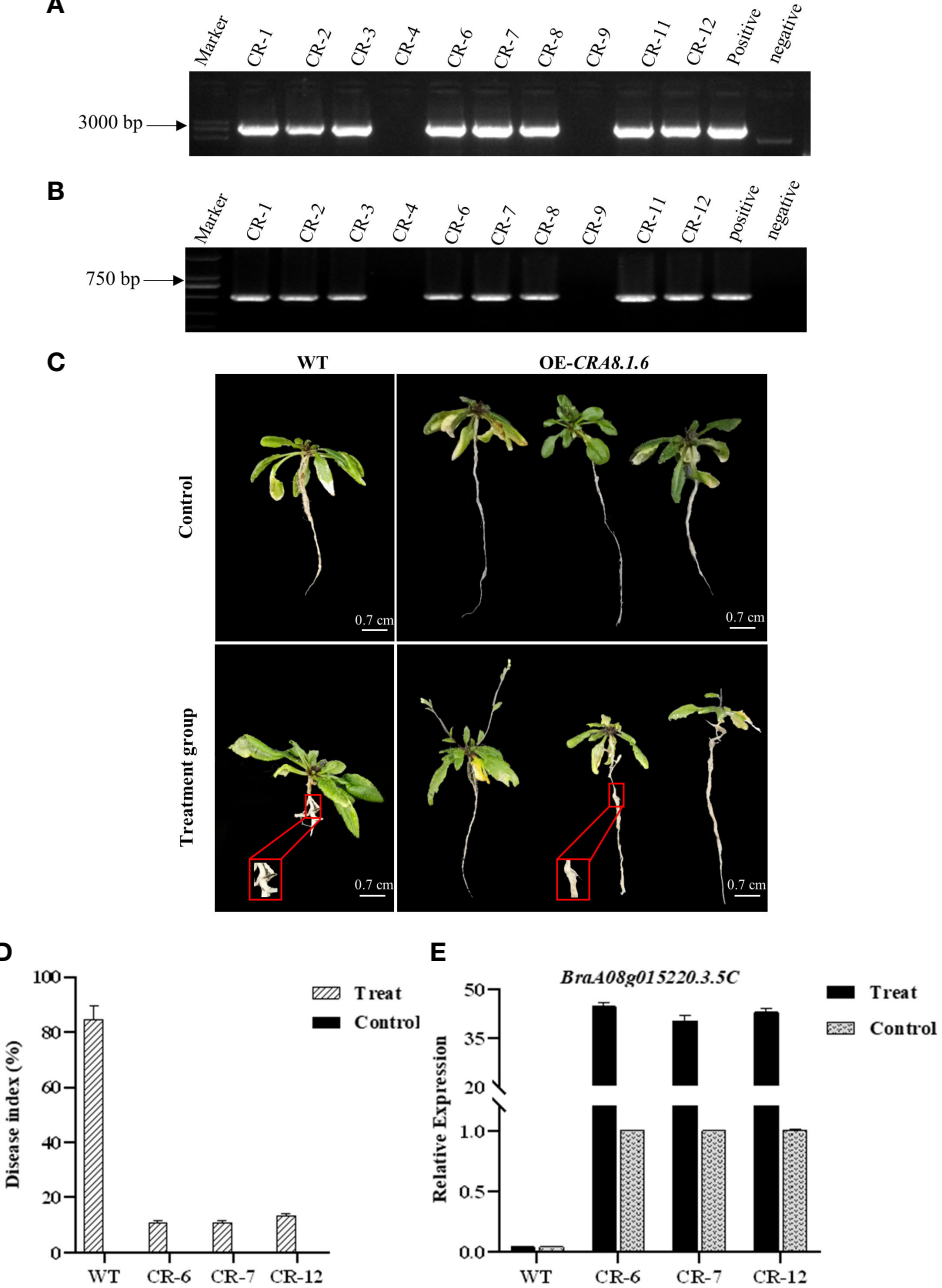
Detection map of genomic primers and hygromycin primers of T_2_ transgenic *Arabidopsis* lines, phenotypic characteristics of overexpressed *Arabidopsis thaliana*, disease index investigation, and qPCR analysis. **(A)** Primer CRA8-full-F/R; **(B)** primer Hyg-F/R. CR1-12: T_1_ lines; positive control: recombinant plasmid; and negative control: H_2_O. **(C)** Disease incidence in wild-type roots and overexpression transgenic *Arabidopsis* following infection with *P. brassicae*. WT, wild-type *Arabidopsis*; OE-*CRA8.1.6*, overexpression *Arabidopsis*. Bar = 0.7 cm, 0.7 cm, 0.7 cm, and 0.7 cm. **(D)** Disease index comparison between wild-type and overexpression transgenic *Arabidopsis* CR-6, CR-7, and CR-12. **(E)** Analysis of candidate gene expression levels in wild-type and overexpression transgenic *Arabidopsis* CR-6, CR-7, and CR-12.

PCR was conducted using specific full-length primers to amplify the target gene from the subclone vector, which has a length of 4,000 bp. The correct cloning vector was digested using *Xho*I + *Eco*RI. Subsequently, the exogenous fragment was ligated into the pBWA (V) HS vector using T4 DNA ligase to obtain the recombinant plasmid (**Supplementary Figure 4**). The recombinant plasmid was confirmed to be correct, and the overexpression vector was successfully constructed. Overexpression *Arabidopsis* lines were identified in hygromycin-resistant medium, and homozygous lines were obtained through consecutive selfing over two generations. DNA extracted from T_1_ transgenic *A. thaliana* was identified (**Supplementary Figure 5**). The recombinant plasmid was the positive control, whereas water was the negative control. The PCR bands observed in the eight transgenic plants were consistent with the bands of the positive control. Consequently, eight T_2_ lines (CR-1, CR-2, CR-3, CR-6, CR-7, CR-8, CR-11, and CR-12) were identified as overexpressing the target gene (**Figures 6A, B**).

### 3.6 Identification of transgenic *Arabidopsis* with target gene expression

Transgenic lines CR-6, CR-7, and CR-12, along with wild-type *Arabidopsis*, were chosen for clubroot phenotypic identification. One set of 25-day-old *Arabidopsis* plants was inoculated with *P. brassicae* spores, whereas the other set was inoculated with water only as an experimental control. The experiments were performed with three biological replicates.

After 30 days of inoculation, the control wild-type and transgenic *Arabidopsis* plants displayed no disease symptoms. In the experimental group, within the overexpression line, CR-6, 22, 5, and 7 out of the 34 plants displayed disease grades 0, 1, and 3, respectively, resulting in a disease index of 10.92%. For the CR-7 line, 19, 5, and 6 out of the 30 plants exhibited disease grades 0, 1, and 3, respectively, with a disease index of 10.95%. Regarding the CR-12 overexpression line, 20, 3, and 9 out of the 32 plants showed disease grades 0, 1, and 3, respectively, yielding a disease index of 13.39% (**Supplementary Table 6**). In wild-type *Arabidopsis*, the frequency of disease stages in CR-6, CR-7, and CR-12 was 5 or 7, with disease rates of 85.71%, 89.01%, and 79.59%, respectively (**Figure 6C**).

The disease index in the overexpression transgenic *Arabidopsis* was significantly lower than that of the wild-type *Arabidopsis* in the experimental group (**Figure 6D**). RNA was extracted from transgenic *Arabidopsis* plants and reverse transcribed into cDNA to analyze the expression of candidate genes. Criterion 1 was used to evaluate the expression level of the candidate genes in transgenic *Arabidopsis* based on their expression levels in the control group. The relative expression of the candidate gene in the wild *A. thaliana* from the experimental and control groups was only 0.04. In contrast, gene expression levels were over 40 in transgenic lines, significantly higher than those in the control (**Figure 6E**).

## 4 Discussion

This study analyzed the clubroot resistance of the inbred line BrT18-6-4-3 and the susceptible DH line Y510. Microscopic observation and qRT-PCR verification of gene expression led us to speculate that the stages between 3 DAI and 9 DAI are crucial for cortical infection and clubroot formation in turnip. The infection cycle of *P. brassicae* typically involves primary infection of the root epidermis followed by secondary infection of cortex tissue. Root hair and cortex infections are thought to occur in host and non-host organisms. We proceeded to morphologically characterize the polymorphic developmental structures of *P. brassicae* during the primary infection in root hairs and epidermal cells, a process that concluded within 7 days of inoculation (Liu et al., 2020a). In this study, we observed that root hair infection occurred in parents at 1 DAI, and swelling of the root of the susceptible parent commenced at 9 DAI. The cortical cells of the susceptible parent harbored numerous spores, whereas those of the resistant parent contained only a few spores. We hypothesize that cortical infection is inhibited in the resistant parent, which is consistent with the study by Liu et al. (2020b).

The *CRA8.1.6* QTL was successfully mapped to a physical interval between 12.255 Mb and 12.365 Mb. Functional annotation of the genes within the QTL suggests that the *BraA08g015220.3.5C* gene is a potential candidate for clubroot resistance. Analysis of gene sequence alignment between lines BrT18-6-4-3 and Y510 revealed 249 SNPs, seven insertions, and six deletions. Among these variations, an LTR retrotransposon (5,310 bp) was identified as an insertion at 909 bp within the first intron. This LTR retrotransposon was not detected in the resistant line; however, another non-functional LTR insertion was detected in our other materials. Therefore, we concluded that LTR insertion may not be responsible for susceptibility.

Additionally, amino acid sequence alignment indicated six non-synonymous SNPs in the TIR domain but had no effect on clubroot resistance. Because these six non-synonymous mutations are present in both resistant and susceptible materials of natural populations. The frameshift mutation that we identified led to early translation termination at 8,551 bp in the susceptible line Y510. This premature termination in the C-terminal LRR domain resulted in a complete loss of function, leading to the loss of clubroot resistance. Recently, the clubroot-resistant genes *Crr1a*, *CRa*, and their alleles have been cloned (Ueno et al., 2012; Hatakeyama et al., 2013, Hatakeyama et al., 2017, Hatakeyama et al., 2022). All of these genes encode nucleotide-binding LRR receptors and feature a TIR domain at the N-terminus. Wang et al. (2023) identified a broad-spectrum clubroot-resistance gene, *WeiTsing*, in *A. thaliana*, which was induced in the pericycle to hinder the colonization of *P. brassicae* in the stele. Moreover, *WeiTsing’s* channel activity is essential for increasing [Ca^2+^]_cyt_ and enhancing plant defense. *WeiTsing* is situated in the endoplasmic reticulum and functions as a calcium-permeable cation-selective channel. However, no *WeiTsing* homologs have been discovered in *B. rapa* or *B. oleracea* (Ochoa et al., 2023). Consequently, current breeding efforts for clubroot resistance in *Brassica* primarily rely on NBS-LRR genes. Hatakeyama et al. (2013) discovered an LTR retrotransposon inserted into the first exon of the susceptible *Crr1a^A9709^* allele, with a similar result observed in Chiifu-401. However, this insertion is not prevalent in CR-resistant Chinese cabbage varieties. Two susceptible *CR* alleles lacking 172 amino acids in the C-terminal region were identified in *A. thaliana*. A chimeric *Crr1a* transgene restored resistance in susceptible *A. thaliana* (Hatakeyama et al., 2022), suggesting that susceptibility is attributed to the absence of the C-terminus.

The candidate gene of *CRA8.1.6* QTL was located in the same region as previously reported *CR* genes: *Crr1^G004^*, *Crr1a^Kinami90-a^*, *Crr1a^Kiko85-a^*, and *Crr1a^Hiroki-b^*. Gene sequencing revealed a candidate *CRA8.1.6* with a genomic length of 4,295 bp and a CDS length of 3,675 bp in the resistant line BrT18-6-4-3. In contrast, in the susceptible line Y510, the genome sequence was 9,586 bp, with a CDS length of 3,644 bp. Sequence alignment of the CDS revealed a 99.4% similarity to *Crr1^G004^* (**Supplementary Table 5**, **Supplementary Figure 4**). We hypothesize that *CRA8.1.6* is likely an allele of the *Crr1^G004^* gene.

The *RPS4*, *RPP1*, and *RPP5* genes in tobacco belonging to the TIR-NB-LRR class R genes are functionally impaired by TIR domain deletion or point mutation. In this study, we aligned the TIR region of the susceptible lines Y510, Crr1a^Kiko85_a^, and Crr1a^Hiroki_b^ with the disease-resistant lines Crr1^G004^ and Crr1a^Kinami90_a^. Six non-synonymous SNPs were identified through amino acid sequence alignment and confirmed to be unrelated to the trait in natural populations. In the susceptible Y510 line, we observed premature translation termination at 8,551 bp and alterations in the LRR domain. The underlying cause could be the insertion and deletion of large fragments in the LRR region, resulting in structural damage to this domain. This mutation results in losing resistance in clubroot (Hatakeyama et al., 2022). We developed two variant-based functional markers, CRA08-InDel and CRA08-KASP1, compatible with genotypes and phenotypes. They showed >90% concordance with the clubroot-resistant phenotype.

## 5 Conclusion

The fine localization distance of *CRA8.1.6* was 110 kb. *BraA08g0152203.5C* is likely the candidate gene for *CRA8.1.6*, encoding a TIR-NBS-LRR protein. Compared to disease-resistant and susceptible materials, we identified 249 SNPs, seven insertions, and six deletions in *CRA8.1.6* (*BraA08g015220.3.5C*). We discovered premature translation termination at 8,551 bp, leading to the loss of the LRR domain at the C-terminus and ultimately resulting in the loss of clubroot resistance. Furthermore, we developed and validated two functional markers for *CRA8.1.6*. This accomplishment represents a significant advancement in molecular research on turnip clubroot resistance.

## Data availability statement

The original contributions presented in the study are included in the article/**Supplementary Material**. Further inquiries can be directed to the corresponding authors.

## Author contributions

XW: Writing – original draft, Writing – review & editing. SX: Writing – original draft, Writing – review & editing. YZ: Data curation, Writing – review & editing. LZ: Data curation, Writing – review & editing. UN: Writing – review & editing. SY: Methodology, Data curation, Writing – review & editing. HS: Project administration, Software, Supervision, Writing – review & editing. WZ: Resources, Writing – review & editing. ZW: Investigation, Writing – review & editing. BT: Formal Analysis, Writing – review & editing. FW: Investigation, Writing – review & editing. YY: Conceptualization, Supervision, Writing – review & editing. XZ: Supervision, Writing – review & editing.
