# Supplementary material for: Fine mapping and candidate gene analysis of CRA8.1.6, which confers clubroot resistance in turnip (Brassica rapa ssp. rapa)
Source: Front Plant Sci. 2024 May 17;15:1355090. doi: 10.3389/fpls.2024.1355090 (PMC11140098; doi:10.3389/fpls.2024.1355090)
Supplement: Supplementary file 1 [file DataSheet_1.zip › Supplementary Material/Supplement Figures.docx]

**Supplementary Figures**


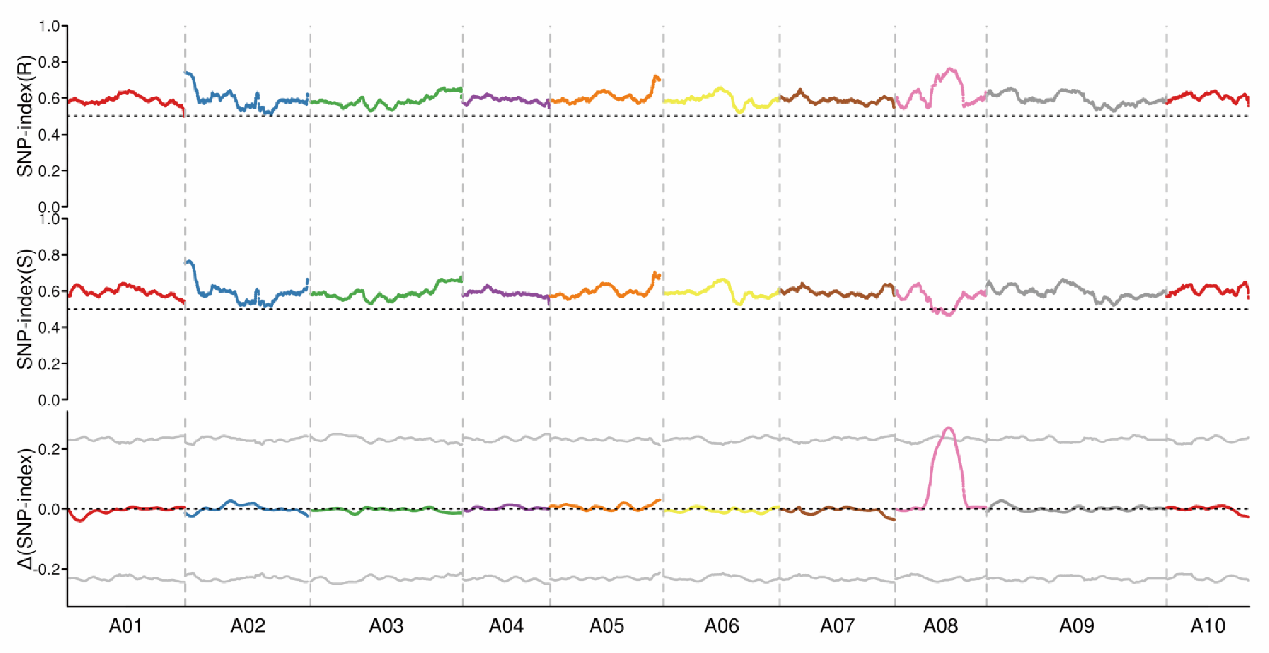


**Figure. S1** Distribution of Δ(SNP-index) values on each chromosome. The candidate interval was located on chromosome A08.

Note: the horizontal gray line is the threshold (the corresponding p value is 1e^-100^); the Δ(SNP-index) value of the pooled pool calculated the proportion of alleles in the parent BrT18-6-4-3.


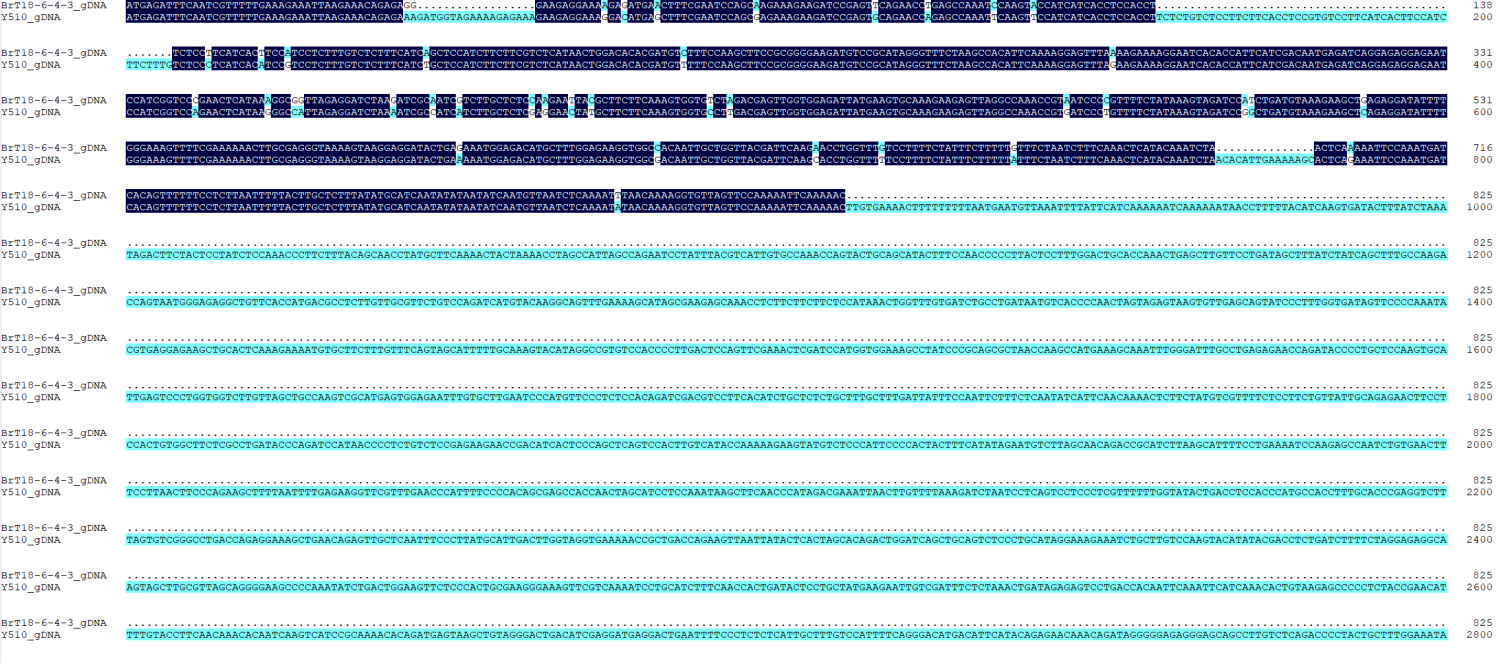

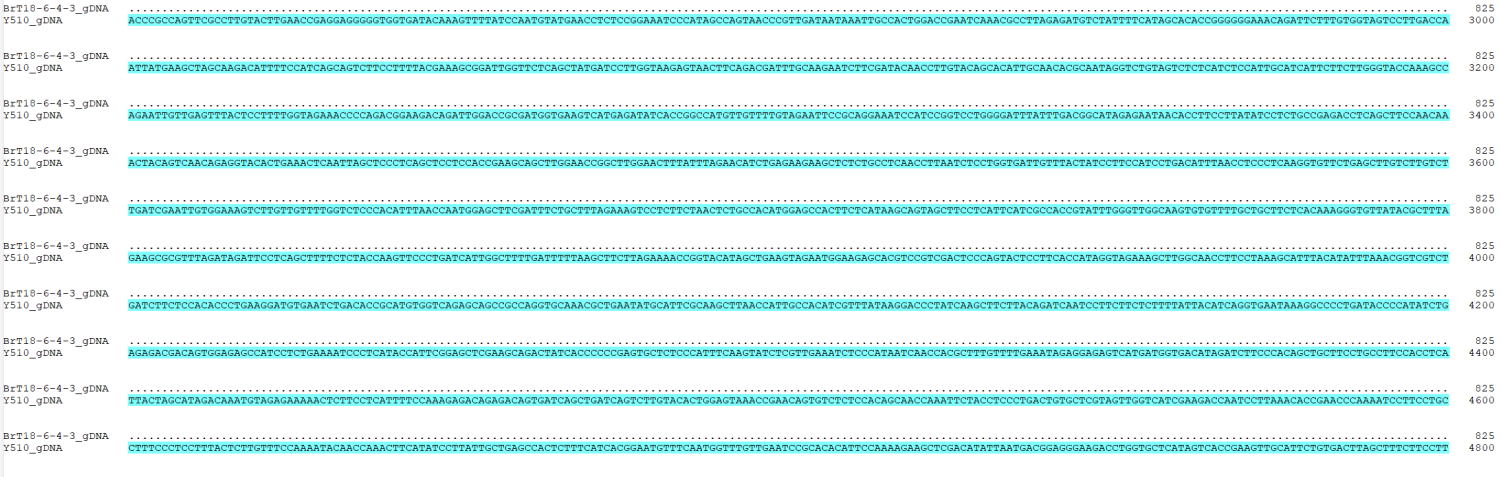

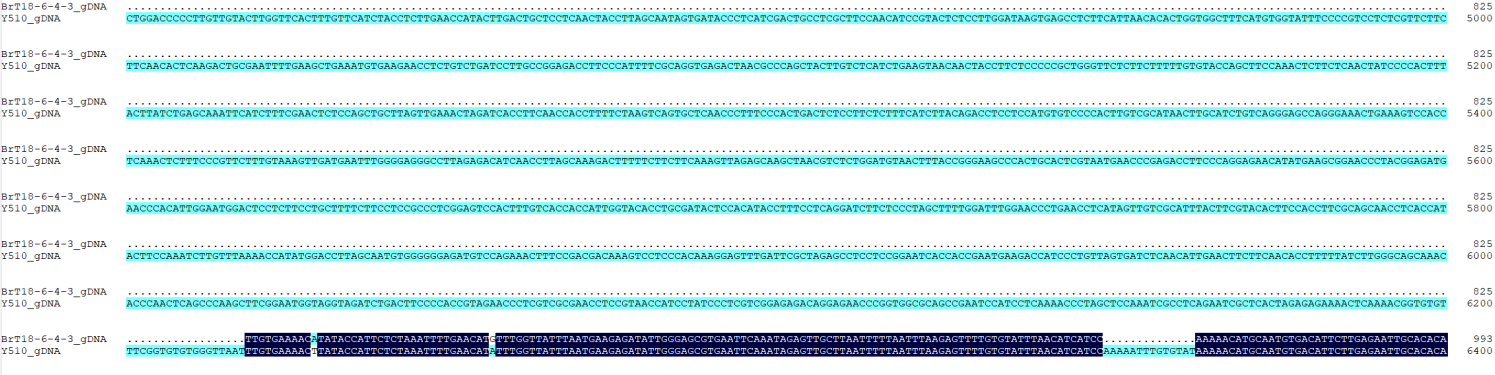

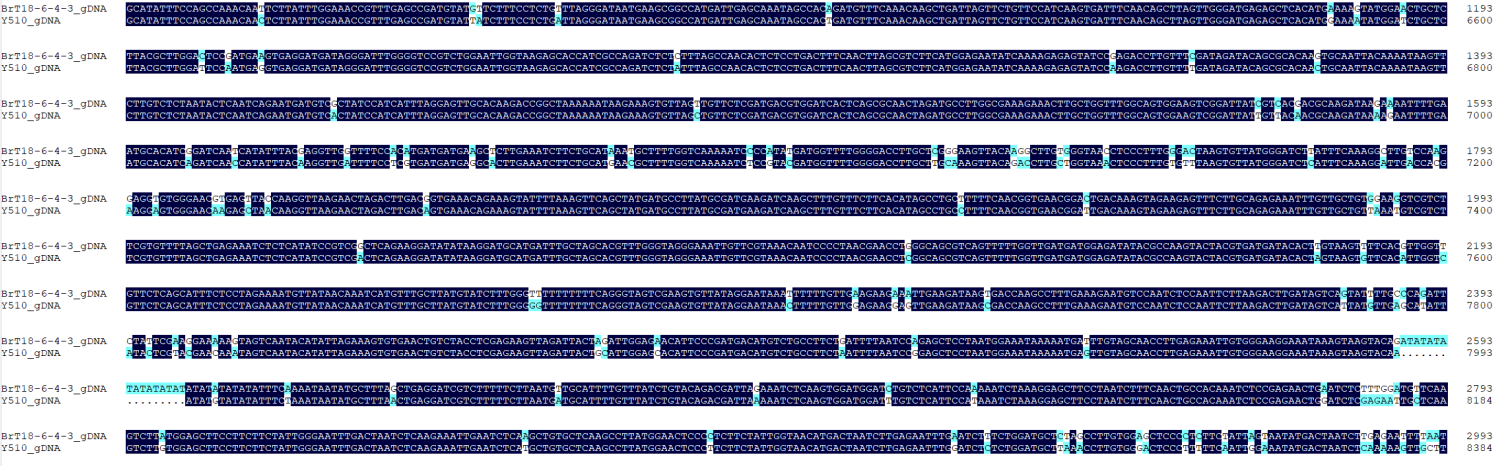

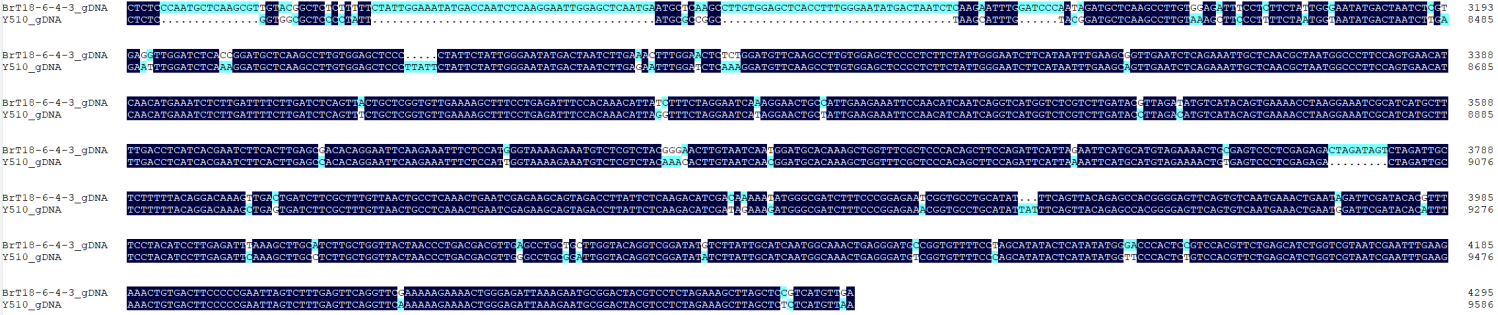


**Figure. S2** Genomic DNA sequence alignment of *CRA8.1.6* (*BraA08g015220.3.5C*) from BrT18-6-4-3 and Y510-9. There were 249 SNP variation and 13 InDel variation between the genomic sequence of BrT18-6-4-3 and Y510.


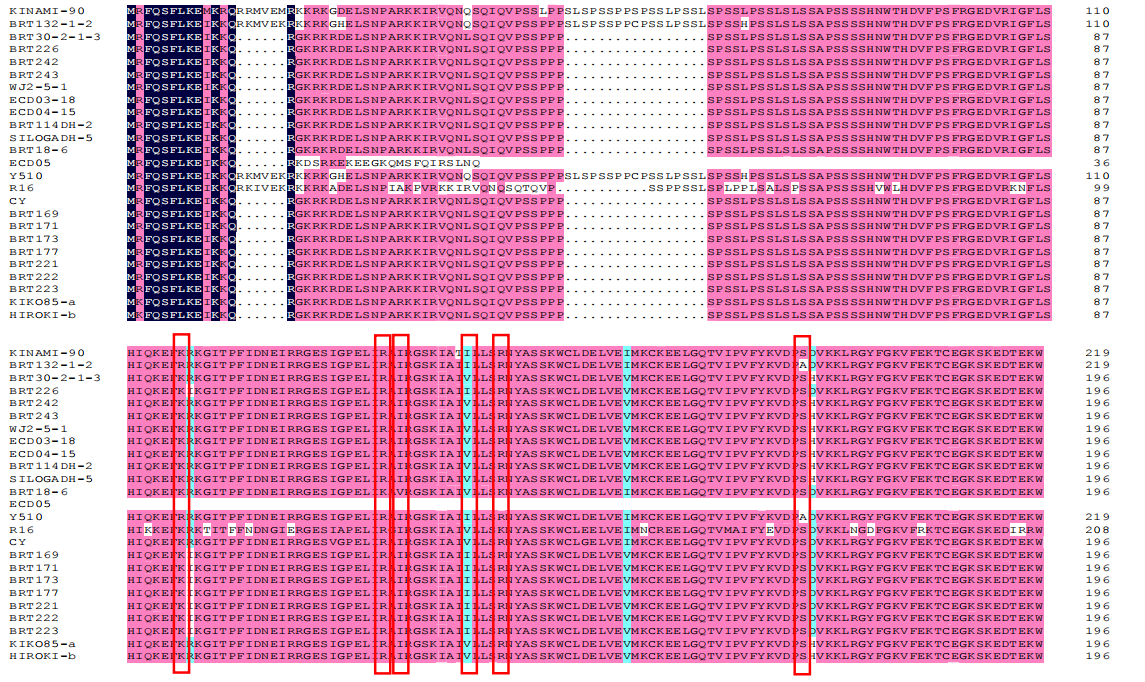


**Figure. S3** Comparison of 6 non-synonymous mutant amino acids in TIR region between 12 resistant materials (KINAMI-90, BRT132-1-2, BRT30-2-1-3, BRT226, BRT242, BRT243, WJ2-5-1, ECD03DH-18, ECD04DH-15, BRT114DH-2, SILOGADH-5, BRT18-6-4-3) and 13 susceptible (ECD05, Y510, R16, CY, BRT169, BRT171, BRT173, BRT177, BRT221, BRT222, BRT223, KIKO85-A, HIROKI-B) materials. In the resistant material BRT132-1-2, the six non-synonymous amino acid mutations were completely contained, but had no effect on its resistance.


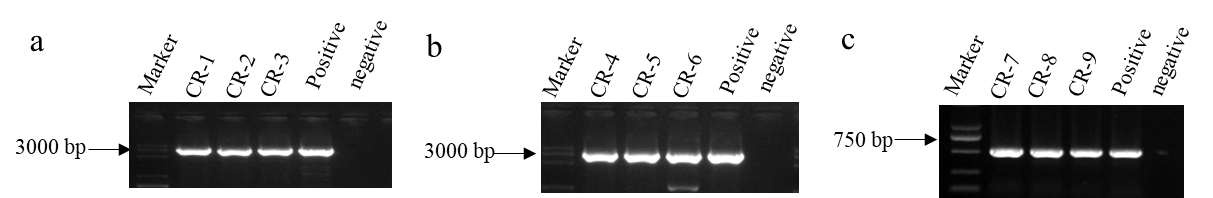


**Figure. S4** Overexpression vector construction diagram. (a) PCR product detection map of E. coli liquid; (b) PCR product detection map of Agrobacterium solution; (c) Recombinant plasmid detection diagram


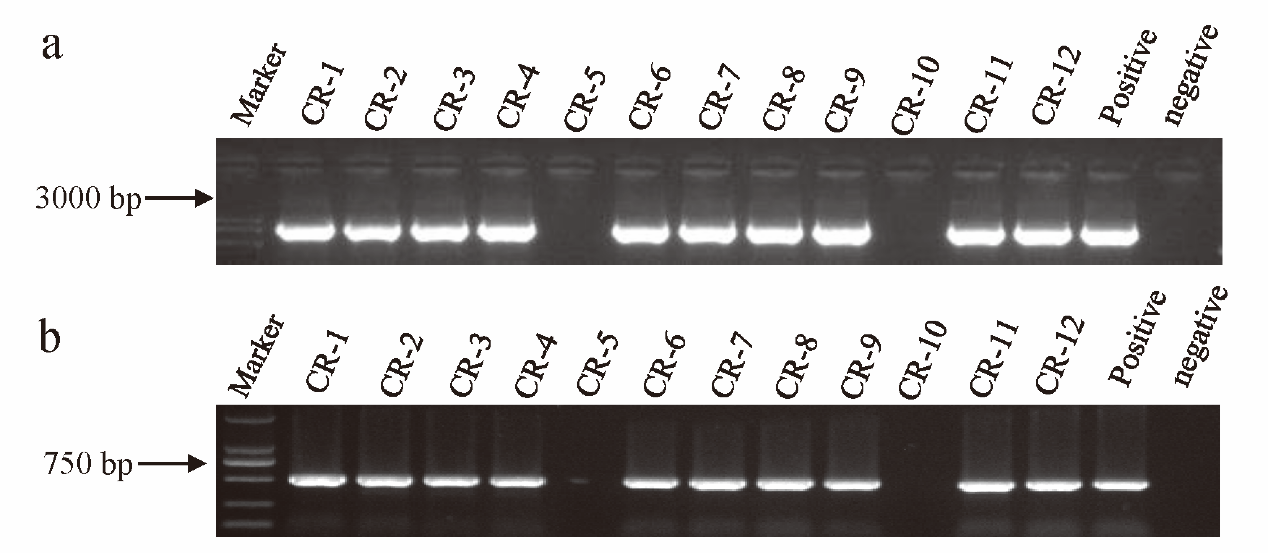


**Figure. S5** Detection map of genomic primers and hygromycin primers of T_1_ transgenic *Arabidopsis* lines

Note: CR1-12: T_1_ *Arabidopsis* lines; positive control: recombinant plasmid; negative control: H_2_O; a: primer CRA8-full-F / R; b: primer Hyg-F / R
